# A systematic review of users experiences of using digital interventions within psychosis: a thematic synthesis of qualitative research

**DOI:** 10.1007/s00127-024-02692-4

**Published:** 2024-05-27

**Authors:** Sophie Dennard, Rupa Patel, Philippa Garety, Clementine Edwards, Andrew Gumley

**Affiliations:** 1 King’s College London, London, UK; 2 University College London, London, UK; 3 University of Glasgow, Glasgow, Scotland

**Keywords:** Psychosis, Qualitative, Technology, Synthesis, Digital Interventions, Interviews

## Abstract

**Purpose:**

Although the development of digital mental health support for people with psychosis has been increasing, the development and opportunities to access this have been more limited compared to other mental health conditions. Qualitative research exploring the experiences of using digital interventions amongst people with psychosis is even less well developed; however, such research is crucial in capturing the experiences of using digital interventions to ensure they are meeting the needs of people with psychosis. This paper aimed to synthesise qualitative data related to the experiences of people with psychosis who have used digital interventions.

**Methods:**

A systematic literature search was conducted of articles published between 1992 and October 2023 using PubMed, MBase, PsycINFO, & OVID Medline. Two reviewers independently reviewed and screened 268 papers. Papers that met inclusion criteria were quality assessed using The Critical Appraisal Skills Programme (CASP) qualitative studies checklist. The Enhancing Transparency in Reporting the Synthesis of Qualitative Research (ENTREQ) checklist was used to guide the structure of the report.

**Results:**

A thematic synthesis of 19 studies revealed six overarching themes which related to different aspects and features of the digital interventions: participants’ relationship with technology; the accessibility of the interventions; how the interventions could impact on individuals’ awareness and management of mental health; enhanced communication and relationships; and opportunities for reflection.

**Conclusions:**

Benefits of using digital interventions are discussed. Areas for development and improvements are highlighted. Finally, recommendations for stakeholders who develop and implement digital interventions for psychosis are made.

## Introduction

Recent research has documented the potential benefits of digital interventions in overcoming barriers to accessing health care [1]. The term ‘digital intervention’ encompasses a wide range of interventions including mobile health (M-Health) interventions that can be delivered via a smartphone or mobile application, via websites or wearable devices, as well as through virtual reality or avatars [2–6].

Smartphone usage among people with psychosis is now at equivalent levels to the general population, with greater interest in developing digital interventions targeting psychosis [7–9]. Previous research has shown that digital interventions for people with psychosis are acceptable and can result in benefits including increased access to services, reduced stigma around receiving mental health treatment, and accommodating difficulties arising from cognitive impairments and/or low levels of motivation [10–16]. However, concerns about security and privacy when accessing support digitally have also been highlighted [17]. Although the growing interest in digital interventions for psychosis is promising, this literature is still relatively sparse in comparison to that concerning other mental health and physical health conditions [18–20].

Currently, qualitative research evaluating digital interventions are lacking in comparison with quantitative studies [21–24]. Existing reviews including qualitative insights have yielded important information regarding how people with psychosis engage with digital interventions [25, 26]. For example, digital interventions tailored to the individual increase the likelihood of people engaging, whilst severe mental health symptoms and technical issues serve as barriers to engagement [26]. It is worth highlighting that Bell et al. did not complete a systematic review of the literature, instead providing an overall synthesis of types of digital interventions and how these can support and benefit people with psychosis. Another review suggested that symptom monitoring and medication adherence tracking were useful features of digital interventions [25]. Although Batra et al. did complete a systematic review, the studies included were a mixture of quantitative or mixed method designs which were then presented through a narrative synthesis [25]. This highlights that previous reviews have provided valuable information, however they did not systematically capture the qualitative experiences of people with psychosis who have completed a specific digital intervention over an extended period of time. Qualitative studies enable us to get an in-depth understanding of participants’ experiences of interactions with, and attitudes and beliefs towards, a particular intervention or phenomenon. Gaining an increased understanding of the experiences of people with psychosis using digital interventions can ensure that they are meeting the needs of those using them and that they can be harnessed as a sustainable way of accessing support.

This review provides a synthesis of studies that adopted qualitative methods to examine the experiences of people with psychosis who have used digital interventions. It aims to provide a comprehensive understanding of how people with psychosis experience using digital interventions including features that may increase engagement and those that act as barriers. Further, it aims to answer the question, how do people with psychosis experience using digital interventions?

## Methods

### Design

This systematic review follows Cochrane guidance on conducting reviews and the Preferred Reporting Items for Systematic Review and Meta-analysis (PRISMA) guidelines [27, 28]. Specifically, the Cochrane guidance for qualitative evidence and the ENTREQ checklist were used to ensure that the review was conducted and reported appropriately [74, 75]. The PICOS framework (participants, interventions, comparators, outcomes, study design) was used to determine the eligibility criteria for study inclusion [29]. This review protocol was registered with PROSPERO, number CRD42022318723 (22/03/2022).

### Inclusion criteria

Studies were included if they had participants who were adolescents and/or adults with psychosis. Studies were included if participants were reported as having severe mental illness (SMI). Studies exploring digital interventions or digitalized forms of assessment that are delivered via smartphone applications and/or web-based interventions that promote mental health, physical health and wellbeing were included. Studies that had qualitative elements which explored the potential effectiveness, usability and acceptability of digital interventions for people with psychosis were included. Only peer-reviewed studies were included.

### Exclusion criteria

Studies were excluded if participants did not have a diagnosis of psychosis, this includes participants that were solely reported to have a diagnosis of bipolar disorder. Studies were excluded if they reported on digital interventions that were evaluating or developed for staff and/or carers of people with psychosis. Studies that reviewed digital interventions that purely provide consultation (telehealth) were not included. Studies that only used quantitative methods to evaluate a digital intervention and papers that report on study protocols were not included.

### Search strategy, data screening and selection

The following databases were searched for this systematic review: PubMed, Embase, PsycINFO, & OVID Medline. Only articles that were published in English and were published between 1992 and October 2023 were included in the search.

Search terms related first to the population comprising Mental health OR Severe mental health OR Severe mental illness OR Psychosis OR Schizophrenia OR Voices OR Hallucinations. The second group of terms related to the digital technologies including Digital health interventions OR Digital interventions OR Digital treatment OR Mobile interventions OR Mobile treatment OR Mobile health applications OR Mobile health apps OR Smartphone OR Smartphone treatment OR Smartphone interventions OR Online treatment OR Online interventions OR Mhealth OR Ehealth. The third term related to experience of technologies including Experience OR User experience OR User perspective OR Engagement OR Satisfaction. The final term related to the methodology including Qualitative OR Interview OR Focus group OR Mixed methods.

**Fig. 1:**
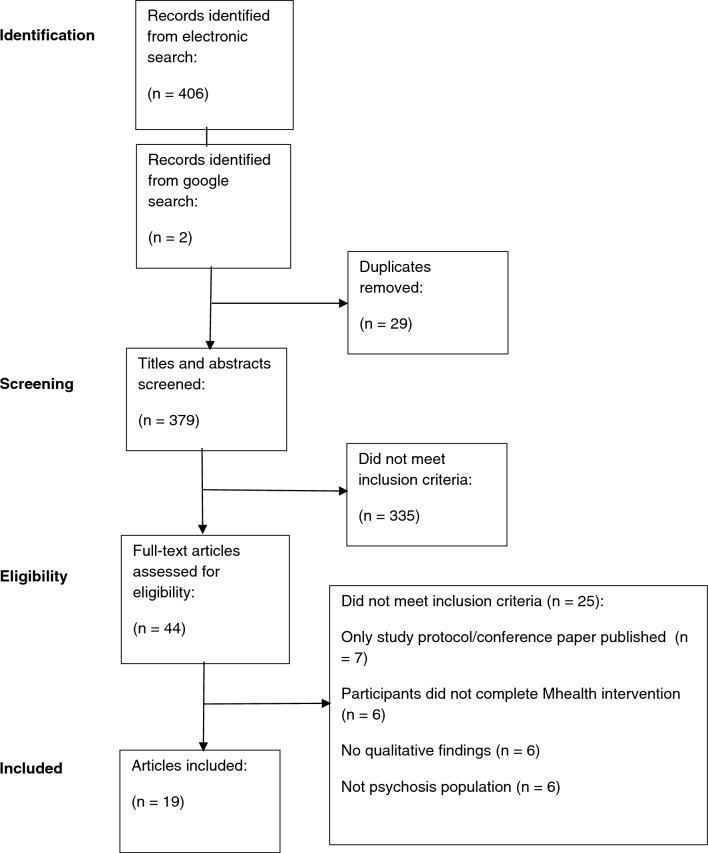
Study selection process

Titles and/or abstracts of studies retrieved using the search strategy were reviewed by the first author (SD) to identify studies that potentially met inclusion criteria. The second author (RP) reviewed the studies to check these decisions. Full texts of potentially eligible studies were retrieved and reviewed by the first author. At least 20% of these studies were independently screened by RP. Final decisions on papers to be included within the review were discussed between SD and RP following this independent review. Both reviewers (SD and RP) extracted data from the papers selected for inclusion in the review. Disagreements or discrepancies between the two reviewers at any stage of the screening and extraction process were resolved through discussion. An Excel spreadsheet was used to record the inclusion decisions and extracted data from the included studies for assessment of study quality. Data extracted from each of the included studies consisted of; aims of the study, ethical issues, study setting, theoretical background of study, sampling approach, participant characteristics, data collection, data analysis approach, key themes highlighted, and data related to the themes. Figure 1 presents the study selection process.

### Assessment of study quality

The Critical Appraisal Skills Programme (CASP) qualitative studies checklist was used to assess the quality of the papers included in this review [30]. The first and second authors (SD and RP) independently assessed the quality of the studies before discussing the quality findings together and agreeing on the quality ratings of each paper. Each item was assigned a score, with ‘1’ indicating yes, and ‘0’ indicating either can’t tell or not addressed. Papers were then given a total score out of 10. Papers with a total score of 9 or above were categorised as being high quality, medium quality papers were those with a total score of 7 or 8, and papers with a total score of 6 or below were categorised as being low quality.

### Data synthesis

The data from the selected studies were analysed using an inductive approach to thematic synthesis [31] in order to provide an understanding of the experiences of people with psychosis using digital interventions. The data synthesis for this current review followed the three stages of thematic synthesis; (1) Individual study findings were treated as raw data within the analysis. Each identified study was coded line by line in order to identify meaning in the data. (2) Codes from across the studies were reviewed together, and those with similar concepts were grouped together and given new, more descriptive names. (3) Final codes were grouped into broader, analytical themes.

### Reflexivity statement

The first author is a White British, female Clinical Psychologist with experience of working clinically with people with psychosis including delivering psychological interventions such as Cognitive Behavioural Therapy for psychosis (CBTp) and Family Intervention for psychosis (FIp). The second author is a female research assistant with an Indian ethnic background who has worked in Early Intervention Psychosis services and Increasing Access to Psychological Therapies (IAPT) services. The remaining three authors are White British Female (PG and CE), and White British Male (AG), all experienced clinical psychology researchers, specializing in working clinically with people with psychosis, developing treatment approaches, including CBTp and digitally supported interventions, and new services. All authors have strong interests in understanding the perceptions and experiences of people who experience psychosis in order to enhance the quality of treatment. As such, the epistemological position of the authors in this review is most closely aligned to a “critical realist” perspective [32].

## Results

### Assessment of study quality

Fifteen out of the nineteen studies were rated as high quality, with a score of 9 or above [12, 33–46]. The rest of the studies were rated as moderate quality, scoring either 7 or 8 [14, 47–49]. None of the included studies were rated as low quality (scoring 6 or below). Scores on individual items for all included studies can be found in Table 1. In all of the studies, there was a clear statement of aims, and the methods were deemed appropriate to address these aims. For five of the studies, the method used to analyse the data was stated, however a more detailed reporting of the steps used to analyse the qualitative data was not clearly stated [14, 42, 43, 47, 49]. The item that was scored most poorly across the included studies was in relation to reflexivity and bias. Only seven out of the nineteen studies had a clear reflexivity statement or explanation of the researchers’ relationship with the participants [12, 33, 34, 39, 42, 44, 45].

**Table 1:** Quality assessment scores of included studies

	Buck et al. 2022	Gowarty et al. 2021	Austin et al. 2021	Bucci et al. 2018	Valentine et al. 2020	Kidd et al. 2019	Lim et al. 2020	Eisner et al. 2019	Naslund et al. 2016	Forchuk et al. 2015	Klein et al., 2019	Meyer et al. 2018	Williams et al. 2018	Schlosser et al. 2018	Palmier-Claus et al. 2013	Greenwood et al. 2023	Lal et al. 2023	Taylor et al. 2022	Zarbo et al. 2022
Was there a clear statement of the aims of the research?	Y	Y	Y	Y	Y	Y	Y	Y	Y	Y	Y	Y	Y	Y	Y	Y	Y	Y	Y
Is a qualitative methodology appropriate?	Y	Y	Y	Y	Y	Y	Y	Y	Y	Y	Y	Y	Y	Y	Y	Y	Y	Y	Y
Was the research design appropriate to address the aims of the research?	Y	Y	Y	Y	Y	Y	Y	Y	Y	Y	Y	Y	Y	Y	Y	Y	Y	Y	Y
Was the recruitment strategy appropriate to the aims of the research?	Y	Y	Y	Y	Y	Y	Y	Y	Y	Y	Y	Y	Y	Y	Y	Y	Y	Y	Y
Was the data collected in a way that addressed the research issue?	Y	Y	Y	Y	Y	Y	Y	Y	Y	Y	Y	Y	Y	Y	Y	Y	Y	Y	Y
Has the relationship between researcher and participants been adequately considered?	?	?	Y	N	Y	?	?	Y	?	?	?	Y	Y	Y	N	Y	N	N	N
Have ethical issues been taken into consideration?	Y	Y	Y	Y	Y	Y	?	Y	Y	Y	Y	Y	Y	Y	Y	Y	Y	Y	Y
Was the data analysis sufficiently rigorous?	?	Y	Y	?	Y	Y	?	Y	Y	Y	Y	Y	Y	?	Y	Y	?	Y	Y
Is there a clear statement of findings?	Y	Y	Y	Y	Y	Y	Y	Y	Y	Y	Y	Y	Y	Y	Y	Y	Y	Y	Y
How valuable is the research?	Y	Y	Y	Y	Y	Y	Y	Y	Y	Y	Y	Y	Y	Y	Y	Y	Y	Y	Y
**Score (out of 10)**	8	9	10	8	10	9	7	10	9	9	9	10	10	9	9	10	8	9	9

Y = Yes,N = Not addressed, ? = Can’t tell

### Thematic synthesis

**Fig. 2:**
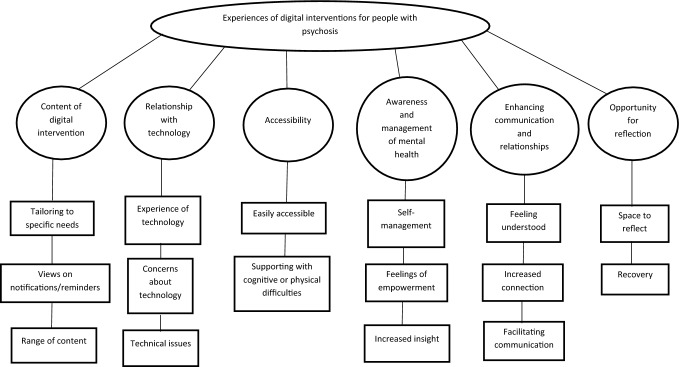
Thematic map of themes and sub-themes

After screening 407 records that were identified in the search, 19 studies were found to meet the inclusion criteria for the thematic synthesis (see Fig. 1).The number of participants across the studies ranged from 6 to 95 (*M* = 22.5). The mean ages of the participants ranged from 20 to 55yrs across the studies. In total, 54.1% of the participants across all of the studies were male, 45.7% were female, and 0.2% were transgender. The ethnicity of participants was not reported in 6 out of the 15 studies. In studies that did report on ethnicity, between 42.1 and 100% of participants were White. 10 out of 15 studies used a mixed-method design, with the remaining four employing only qualitative methods. Further information on the included studies are detailed in Table 2. The synthesis resulted in six overarching themes and sixteen sub-themes (Table 2). The six themes were: (1) Content of digital intervention, (2) relationship with technology, (3) accessibility, (4) awareness and management of mental health, (5) enhancing communication and relationships, and (6) opportunity for reflection. A thematic map describing the themes and sub-themes can be found in Fig. 2 (Table 3).

**Table 2:** Summary of studies included in review

Study authors and type of digital intervention	Study population and number of participants (N)	Participant demographics	Study setting	Study method, qualitative data collection and analysis	Sampling strategy	Summary of findings
Buck et al. 2022; FOCUS mHealth app; pilot feasibility study; United states	Severe mental illness (17)	Age, mean 55.12; Gender, 71% female, 29% male; Ethnicity, 65% White, 18% Black or African American, 12% Asian, 6% American Indian or Alaskan Native	Outpatient mental health clinic	Mixed method; Semi-structured interviews; No specific data analysis approach reported	Convenience sampling	**Main themes:** -Easy access to self-management tools that were consistently available -Ability to engage in reflection and self-management -Opportunity to identify specific coping skills to deal with symptoms in the moment -FOCUS not always meeting individual needs -Feeling bothered by notifications -Needing to pay close attention to phone -Desire for FOCUS to be more integrated into routine services and care
Gowarty et al. 2021; QuitGuide, quitStart apps; Usability and acceptability study; United States	Severe mental illness (17)	Age, mean 29; Gender, 59% male, 41% female; Ethnicity, 94% White	Community mental health centre	Mixed method; Semi-structured interviews; Thematic analysis	Convenience sampling	**Main themes:** -Ease of use for QuitGuide app -Initial difficulty with navigation quitSTART app -Benefits of a positive and supportive tone of app -Feeling cared for -Ability to complete daily tracking of cigarettes smoked -Repeated reminders of lack of progress impacting on ability to quit -Desire for more notifications as reminders to use the app -Desire for an enhanced tracking feature -Desire for a chat feature to enable connection with others -Desire for more opportunity to enter free-text responses
Austin et al. 2021; IMPACHS mHealth solution; user experience study; Denmark	First episode psychosis (10)	Age, mean 24.06; Gender, 75% female, 25% male; Ethnicity, not reported	Early intervention in psychosis service	Mixed method; Semi-structured interviews; Thematic analysis	Convenience sampling	**Main themes:** -Easily accessible -Supporting memory to complete tasks and recall events -Promoting conversations with therapists -Building a shared understanding with therapists -Opportunity for reflection on mental health experiences -Factors affecting engagement including presence of negative symptoms, Bluetooth impacting on their paranoid ideation, and difficulties logging on to the solution
Bucci et al. 2018; Actissist; Proof-of-concept RCT; United Kingdom	First episode psychosis (15)	Age, mean 20.21; Gender, 62.5% male, 37.5% female; Ethnicity, 87.5% White British/Irish, 8.3% Black Caribbean/African, 4.2% Asian	Early intervention in psychosis services	Mixed method; Semi-structured interviews; Thematic analysis	Convenience sampling	**Main themes:** -Ease of access -Inspires confidence and empowerment -Facilitates self-management -Becomes part of your routine -Desire for less repetition -Need for increased opportunity to personalise content
Valentine et al. 2020; Horyzon’s; qualitative study following on from RCT; Australia	First episode psychosis (12)	Age, 19 to 28 (mean 23); Gender, 58.3% female, 41.6% male; Ethnicity, not reported	Early intervention in psychosis service	Qualitative; Semi-structured interviews; Interpretative phenomenological analysis	Purposive sampling based on overall engagement levels	**Main themes:** -Shared experience as the catalyst for a cocreated social space -The power of peer support -An upbeat environment -Interruptions to being in the Horyzons space including lack of motivation, feeling self-conscious and experiencing symptoms of paranoia and social anxiety
Kidd et al. 2019; App4Independence (A4i); Feasibility study; Canada	Schizophrenia spectrum, primary psychotic disorder (38)	Age, 19–61 (mean 31.42); Gender, 71.1% male, 26.3% female, 2.6% transgender; Ethnicity, 42.1% White, 23.7% Black of African or Caribbean origin, 23.7% Mixed, 10.5% other	Outpatient mental health clinic	Mixed method; Check-in contacts, semi-structured interviews; Content analysis	Convenience sampling	**Main themes:** -Scheduling and reminder functions seen as positive -Availability of strategies to manage their symptoms -Opportunity to have peer-to-peer connections through a community space -Ability to record notes for doctors -Desire for customisability -Increased opportunity to provide detailed feedback -Concerns about being monitored
Lim et al. 2020; + Connect, pilot study; Australia	First episode psychosis (12)	Age, 17–25 (mean 20.5); Gender, 75% male, 25% female; Ethnicity, 66.7% White, 25% Asian Australian or Asian, 8.3% African Australian	Early intervention in psychosis service	Mixed-method; Semi-structured interviews; Content and thematic analysis	Purposive sampling of patients from a community early intervention service who had a score of > 38 on the UCLA Loneliness scale and who had expressed a desire to connect with others	**Main themes:** -Content of app east to relate to -Evidence of positive change over time -Notifications helpful as reminder to use app -Increased feelings of positivity -Increased confidence and opportunity to connect with others -Providing a space to reflect on previous experiences -Easy to use -Tasks putting people out their comfort zone
Eisner et al. 2019; ExPRESS, feasibility study; United Kingdom	Schizophrenia spectrum (16)	Age, mean 37.9; Gender, 67% male, 33% female; Ethnicity, 83% White, 11% Black or Black British, 6% Asian or Asian British	Acute inpatient units and community based mental health teams (e.g. Community Mental Health Teams, Early Intervention Teams, Assertive Outreach Teams, Crisis Teams)	Qualitative; Semi-structured interviews; Framework analysis	Convenience sampling	**Main themes:** -Being able to connect with others -Easy to use -Increased opportunity to reflect on experiences and notice changes in symptoms over time -Ability to detect symptoms early -Opportunity to normalise experiences and build hope for recovery -Completing app feeling like a chore -Increasing honesty about experiences -Barriers to engagement including high levels of positive symptoms, using a study phone increased chances of forgetting, and lack of literacy
Naslund et al. 2016; Fitbit Zip; acceptability study; United States	Severe mental illness (11)	Age, mean 48.2; Gender, 73% female, 27% male; Ethnicity, 100% non-Hispanic White	Community mental health service	Mixed-method; Semi-structured interviews; Content analysis	Purposive sampling of patients in a community mental health service who had enrolled in a 6-month lifestyle intervention	**Main themes:** -Fun and motivating way to reach goals -Easy to use -Ability to see proof of physical activity -Ability to have easy access to data stored in one place -Concerns about inaccurate information -Limited experience of mobile technologies impacting on engagement -Desire for more instruction and support using technology
Forchuk et al. 2015; Mental Health Engagement Network (MHEN), Lawson SMART record (LSR); user experience study as part of an RCT; United Kingdom, Canada	Psychosis (95)	Age, 18–80; Gender, 58.9% male, 41.1% female; Ethnicity, not reported	Community mental health services	Mixed-method; Focus groups; Leininger's qualitative data analysis	Convenience sampling	**Main themes:** -Versatile functionality -Ability to stay connected with others -Feeling safer and more connected -Increased self-awareness and ability to track moods -Lack of understanding of technology a barrier to use -Difficulty keeping track of log in information -Overwhelming amount of information -Desire for simplification -Desire for appointments in app to be linked with phone calendar
Klein et al. 2019; Kick.it app; feasibility and acceptability study; Australia	Severe mental illness (12)	Age, 31–53 (mean 47.5); Gender, 67% Male, 33% female; Ethnicity, not reported	Community mental health service	Qualitative; Semi-structured interviews; Thematic analysis	Purposive sampling of patients in a community mental health team who had attempted to give up smoking in past 12 months, or who had previously smoked	**Main themes:** -Desire for more specific tailoring of apps to meet psychosocial needs -Increased availability of practical solutions that can be accessed in real time -Decreased reliance on memory for recall -Receiving positive messages are useful and supportive -Having support available at the touch of a phone -Limited knowledge in technology seen as barrier when navigating the app -Desire for space where can feel cared for and not judged -Desire to have feature where can connect with others
Meyer et al. 2018; Sleepsight platform; feasibility, acceptability and user experience study; United Kingdom	Schizophrenia (14)	Age, 30–54 (mean 44.1); Gender 60% male, 40% female; Ethnicity, not reported	Community mental health service	Mixed-method; Semi-structured interviews; Grounded theory approach	Convenience sampling	**Main themes:** -Easy to use -Ability to detect relapses earlier -Personalised messages increasing motivation -Having a wearable device supported attaining goals -Symptom diary increased insight and reflection on symptoms -Symptom diary can become tedious over time -Concerns about false alarms -Lower adherence when symptom levels are higher
Williams et al. 2018; Self-Management and Recovery Technology (SMART); user experience study; Australia	Psychotic disorder (6)	Age, 19% under 30, 39% 30–45, 42% over 45; Gender, 67% female, 33% male; Ethnicity not reported	Community mental health service	Qualitative; Semi-structured interviews; Constructivist grounded theory	Purposive sampling of patients in community mental health settings who had already used the SMART website in routine practice	**Main themes:** -Helping to feel less alone -Increased feelings of connection -Feeling inspired by others -Opportunity to learn new information -Enhancing meaning of, and hope for recovery -Feeling drained hearing about others' experiences -Wanting to have distance from mental illness -Limited access to technology impacted on engagement
Schlosser et al. 2018; PRIME app; RCT; United States	Severe mental illness (38)	Age, mean 24.32; Gender, 60% male, 40% female; Ethnicity, 50% White, 15% Asian, 12.5% African American, 22.5% Other	Mixed community settings	Mixed-method; Semi-structured interviews; No specific data analysis approach reported	Convenience sampling	**Main themes:** -PRIME app was acceptable -Helped in feeling less alone -Easy to access support when needed -Increased feelings of hope and connection -Decreased feelings of suicidality and helplessness
Palmier-Claus et al. 2013; Text message and Android smartphone app; Randomised repeated-measure cross-over design study; United Kingdom	Non-affective psychosis (24)	Age, mean 33.04; Gender, 76% male, 24% female; Ethnicity, 70.8% White British, 8.3% Mixed-Race British, 8.3% Black British, 4.2% Black African, 4.2% Black Caribbean, 4.2% White Other	Mixed community settings	Qualitative; Semi-structured interviews, Framework analysis	Convenience sampling	**Main themes:** -App was quick and easy to use -Could be integrated in day-to-day routine -Limited questions could feel repetitive over time -Potential for more immediate support -Enhanced care from clinicians -Usability impacted by technological difficulties -Greater concentration on symptoms
Greenwood et al. 2022; SlowMo ‘webapp’; RCT user experience study; United Kingdom	Psychosis (22)	Age, mean 44.9; Gender, 82% male, 18% female; Ethnicity, 82% White British	Community mental health services	Qualitative; Semi-structured interviews; Thematic analysis	Consecutive and purposive sampling of participants who had completed at least one SlowMo session and a 24-week follow-up	**Main themes:** -Opportunity for help and learning new skills -Feeling listened to -Therapist crucial to therapy and access to technology -Connecting with others reduced isolation -Blended therapy can be therapeutic, but sometimes overwhelming -Lack of interest or technical issues with app impacts engagement -Therapy helped to reduce paranoia, increase confidence and engagement with activities
Lal et al. 2023; HoryzonsCa; Pilot study; Canada	First episode psychosis (20)	Age, mean 26.8; Gender, 52% female, 43% male, 4% other; Ethnicity, 52% White, 17% Black, 13% Latin American, Other 22%	Early intervention clinic	Mixed methods; Qualitative feedback questionnaire; No specific data analysis approach reported	Purposive sampling based on minimum log-in rates	**Main themes:** -Difficulty interacting with others -Easy to understand and use -Preference for an app-based intervention -Being able to track moods and progress would be beneficial -Making content more interactive could increase engagement -Desire for more information about the website -Valuing a confidential and protected space
Taylor et al. 2022; Feasibility and acceptability study; CBTi for sleep difficulties (ExpiWell); United Kingdom	Psychosis (10)	Age, mean 35.57; Gender 64.29% male, 35.71% female; Ethnicity, 42.86% Black/African/Caribbean/Black British, 21.43% White, 7.14% Any other ethnic group, 14.29% Asian/Asian British, 14.29% Mixed/Multiple Ethnic Groups	Early intervention service, Community mental health team	Mixed methods; Semi-structured interview; Thematic analysis	Convenience sampling	**Main themes:** -Helpful in improving sleep -Learning new strategies beneficial -Easy to fit app into daily routine -Daily reminders helped to increase engagement -Ability to contact therapist for support beneficial -Technical issues could impact on ability to engage -Too much written content seen as negative -Desire for more variety in content and increased personalisation -Mood could impact on engagement
Zarbo et al. 2022; Usability study; DiAPAson project; Italy	Schizophrenia spectrum disorders (42)	Age, mean 43.48; Gender, 57.05% male, 42.95% female; Ethnicity, not reported	Inpatient service, Outpatient clinic	Mixed methods; Semi-structured interview; Interpretative Phenomenological Analysis	Convenience and snowball sampling	**Main themes:** -Increased self-reflection and self-confidence -Increased feelings of being supported -Ability to support research and help themselves increased engagement -Complex questions affected engagement -Amount of notifications and interruption to daily life impacted on engagement -Desire to write free text and personalise notifications

#### (1) Content of digital intervention

This theme relates to specific features of the digital interventions that participants found helpful and unhelpful, and also suggestions on how the content of the digital intervention could be improved. This theme is comprised of three subthemes: (I) tailoring to specific needs, (II) views on notifications/reminders, and (III) range of content. For participants, having interventions that felt meaningful to their experiences as well as feeling they can trust the information within interventions are important for motivation and longevity of use. Notifications received through interventions can be helpful, encouraging engagement when it fits within daily routines and other activities. When notifications are inaccurate, causing additional stress, they can reduce people’s willingness or motivation to use the intervention. This may lead to participants ignoring notifications which could impact on long term usage or effectiveness of interventions. When tasks feel mundane or participants no longer feel they are able to learn new skills, interest in or motivation to use interventions may decrease. There, however, needs to be a balance between feeling challenged with learning new skills, however not having content that is too challenging which could cause people to feel overwhelmed or deskilled. Providing solution focused interventions can help participants to feel good and think more positively about the intervention, ensuring that it feels like a positive addition to their lives.

(I) Tailoring to specific needs

Being able to customise and tailor interventions to individual needs was reported as an important feature. This included being able to see visual representations of their own experiences, personalising reminders, and having the option to write qualitative feedback [33, 35, 40, 43, 46–48]. ‘’I would've changed my prompts to check in with my sleep’’ [p.6, 47]. Participants reported difficulty using interventions in instances where the content was hard for them to relate to, or when there were concerns related to the accuracy of data recorded in the intervention [36, 37, 43, 47]. ‘’Hard to relate to the content, mostly for teenagers (had to put examples to my own context)’’ [Participant 6, Suppl material, 48].

(II) Views on notifications/reminders

Notifications and reminders were consistently reported as helping participants to complete tasks such as remembering to take medication, or feeling supported when receiving positive messages [12, 33–36, 45, 47]. ‘’most participants in both groups commented on the overall positive tone of the apps and how this was a necessary attribute to maintain their engagement over time’’ [p. 9, 36]. Negative views associated with notifications and reminders included receiving too many prompts, timing inaccuracies, and receiving false alarms on potential relapses [14, 33, 39, 47]. ‘’Overall, 5 participants commented on how they would have concerns over false alarms—for example, whether sleeping poorly in the absence of deterioration in symptoms would trigger a response’’ [p. 8, 39].

(III) Range of content

The content range of interventions were reported to be too limited, with participants expressing a need for the content to be broader and to have more options. This was especially the case after a sustained period of continued use, which could be seen as repetitive over time [33, 39, 44–48]. ‘’..I found content on the actual app was too limited..” [Participant 128, p.1078, 14]. Participants also commented on positive aspects of the intervention content including finding it enjoyable answering questions, the overall supportive tone, and having more solution-focused content [34, 38, 44, 47]. ‘’Audrey identified the positive environment on Horyzons as an important element of her high usage level. She did not want to spend time at a place where “the people are all negative” [p. 7, 44].

#### (2) Relationship with technology

This second theme is comprised of three subthemes which include participants’ views on technology and their previous experiences of using technology. These were (I) experience of technology, (II) concerns about technology, and (III) technical issues. Existing experience or views about technology could impact on people’s initial willingness or ability to use interventions, however these views can be altered through support from others and with increased familiarity. Having a lack of experience using technology could increase feelings of disempowerment, disconnection and confidence. This could mean that participants do not experience the full benefits that such technology could offer. Technology could impact or interact with pre-existing concerns about monitoring and therefore impact on paranoia. The way in which tracking or monitoring is perceived could impact on how attitudes towards interventions which could be negative or create a sense of safety from knowing others may be supporting from afar.

(I) Experience of technology

A lack of experience using technology was cited as a reason why participants found it difficult to engage with an intervention, however for some, this improved over time [33–35, 46]. ‘’..the rest of us never had a smartphone. I think a class on how to use it would be helpful’’ [Participant 6, p.10, 40]. For others, this lack of experience could restrict their interaction with the intervention and affect their motivation to use it, or in some cases, result in them discontinuing their use [12, 34, 35, 38, 43]. ‘’Lack of smartphone experience was a barrier in some cases, with 1 participant accidentally deleting the app from the phone and 2 others commenting that their lack of smartphone experience prevented them from accessing the app's extra features’’ [p. 17, 34].

Receiving training and/or support with using the intervention was, in some instances, helpful and enabled people to continue using it even after experiencing initial difficulties. However, limited access to the internet could cause some participants to continue experiencing difficulties in engaging [33, 40, 43, 45]. ‘’watching the videos at home supported the information to “sink in” for Viv. Participants who had no or limited Internet access at home due to reception, accommodation, or financial difficulties, did not have this opportunity’’ [p. 8, 45].

(II) Concerns about technology

Concerns about technology, specifically with privacy and access to data were raised across a number of studies [33–35, 37–39, 44]. Some participants reported experiencing feelings of paranoia about being monitored or concerns that others would be able to see the content of the intervention whilst using it in public [12, 34, 39, 46]. ‘’It's difficult for me … yeah I always felt a bit of conscious somebody might be coming along and looking over your shoulder'' [Participant S8, p. 690, 12]. For others, being monitored or tracked by therapists was seen as a positive, leading them to feel safe and supported [33, 43]. ‘’participants were positive about the therapist being able to track how they were doing’’ [p. 947, 33]. Receiving further information regarding privacy settings and confidentiality was reported to help participants feel more comfortable engaging with the technology and disclosing their personal experiences [38, 48]. ‘’most participants liked the inclusion of terms and conditions that outlined the privacy settings and rules of use to alleviate potential concerns around engaging with the social network’’ [p. 10, 38].

(III) Technical issues

Technical issues associated with the digital interventions were reported in a number of studies including reduced battery life, compatibility issues, difficulty navigating apps, or connecting to Bluetooth [33–36, 40, 43, 47, 48]. ‘’Just the amount of work you had to do to get a quick reminder of what you wanna do […] you'd have to go through quite a bit just to get to the section that you want’’ [Participant 3, p.729, 43]. Experiencing such difficulties caused some participants to feel discouraged and frustrated having to spend time to fix them [40]. ‘’It was kind of a pain in the neck so I got discouraged with that. It wasn’t the phone; it was because I didn’t know the phone.” [p. 9, 40].

**Table 3:** Synthesis themes and participant quotes

Themes	Sub-themes	Participant quotes
*Content of digital intervention*	Tailoring to specific needs; views on notifications/reminders; range of content	“…It seemed like it’s prompted me too often.’’ (Participant 128, p.1078, Bucci et al., 2018) “..Helped me be more positive and helped me realise my moods, and helped remind me to take my meds..’’ (Participant 12, p.8, Buck et al., 2022) “Keep a journal so that they (young people) know how they’re feeling (they keep track of everything)’’ (Participant 10, Suppl material, Lal et al., 2022) “Some commented that the ambient sound detector wasn’t necessary since they didn’t have hallucinations some noted that scheduling and notes to doctor functions weren’t relevant for them’’ (p. 12, Kidd et al., 2019) “Have more solutions, more things going on. More content. Maybe for PTSD’’ (p. 8, Buck et al., 2022; Klein et al., 2019) “It kept giving me badges that I didn’t do…It gave me one at seven days smoke free, which I wasn’t, even though I was trying not to smoke’’ (p. 10, Gowarty et al., 2021) “Clients suggested that both the appointment reminders and tracking functions within the LSR were useful’’ (p. 5, Forchuk et al., 2015) “These messages could also be annoying if he was using his phone or otherwise occupied’’ (p. 948, Austin et al., 2021 “I put it in for a reminder and it emails at the beginning of the day. Well, that doesn’t help remind… [me] to take my bedtime meds” (p. 7, Forchuk et al., 2015) “6 participants stated that the symptom diary became repetitive and tedious’’ (p. 9, Meyer et al., 2018) “I did enjoy answering the questions… I didn’t see it as a chore’’ (p. 16, Eisner et al., 2019) “Not really anything to enjoy actually doing the app… It’s not like you’re playing a computer game or you know’’ (p. 16, Eisner et al., 2019)
*Relationship with technology*	Experience of technology; concerns about technology; technical issues	“..Participants reported feeling occasionally suspicious toward the technology and worried that their personal information was at times being monitored’’. (p.8, Meyer et al., 2018) “All users indicated that a variety of factors could affect their engagement with the solution….issues relating to difficulties in navigating in the mHealth solution’’ (p. 947, Austin et al., 2021) “Using the website with the worker who supported participants to “get the hang of it” (Melanie), “remind(ed) me how to find certain things” (Bill), or “prompted me to put something (my views) down’’ (p. 6, Williams et al., 2018) “Yeah, I think you could have a little class to show us how to use them, do some exercises or stuff’’ (p. 10, Naslund et al., 2016) “Patients reported that sometimes they did not know how to correctly perform, in particular in relation to the use of the mobile application (i.e., how to open the app, or how to solve problems) and felt worried in relation to its use. However, in most cases, an early feeling of inadequacy changed into improved self-confidence” (p. 5, Zarbo et al., 2022) “Participants indicated that increased interactivity and therapist guidance could have been beneficial” (p. 727, Taylor et al., 2022) “So yeah, because I’m, I’m not a technical person at all […], that’s the only downside of it is if it doesn’t work okay, then it, it has quite an impact” (Participant 6, p. 729, Taylor et al., 2022) “Never used a computer, but I learnt with [therapist]” (Participant L21, p. 688, Greenwood et al., 2022) “I have bought things like iPod’s and Nintendo but I know the main thing is the smart phone but it’s too much technology for me. I can’t really be bothered to take the phone out with me” (Participant S9, p. 690, Greenwood et al., 2022) A group at a certain date where you talk about the website. A mention it is safe and confidential. (Participant 9, Suppl material, Lal et al., 2023) “I just think about the other person on the other side having to sit there and read the questions. How do they work it out?’’ (p. 18, Eisner et al., 2019) “This individual noted anxiety regarding text messages which made this person feel that someone was “monitoring” her’’ (p. 12, Kidd et al., 2019) “You could say that, when I was at my lowest, I didn’t use the app (...) I was afraid it would become worse’’ (p. 948, Austin et al., 2021) “Generally, users felt that the wearable device would be better tolerated in this scenario than the smartphone diaries’’ (p. 9, Meyer et al., 2018) “One participant reported intermittent concern over radiation emerging from the optical heart rate monitor’’ (p. 8, Meyer et al., 2018) “…Persecutory thinking (i.e., “Something that follows me everywhere and alerts me”, “It videotaped everything I said”)” (p. 5, Zarbo et al., 2022) “The fact that there’s an app, and there’s someone like [trial therapist] to like, keep me in tack or like keep checking up on my progress. I think those, those elements made the experience better for me” (Participant 7, p. 728, Taylor et al., 2022) “I find I have to charge my phone all the time” (p. 8, Forchuk et al., 2015) “Make this app available for iPhone’’ (p. 6, Buck et al., 2022) “One participant linked the Bluetooth problem directly to his paranoid ideation, where he believed an external entity was controlling his phone and trying to monitor his behaviour’’ (p. 948, Austin et al., 2021) “Login to use (another username + password to remember)” (Participant 15, Suppl material, Lal et al., 2023)
*Accessibility*	Easily accessible; supporting with cognitive/difficult difficulties	“..It was perfect, it’s like an immediate help’’. (Participant 106, p.1078, Bucci et al., 2018) “It’s like a 34/7 therapist in my pocket.’’ (Participant 11, p.6, Buck et al., 2022) “Some [challenges] were hard. Yeah, some were tricky.’’ (p.883, Lim et al., 2020) “People with SMI can use their phone anytime and anywhere to connect with other people who are also using the app’’ (p. 10, Klein et al., 2019) “Among quitSTART users, difficult navigation was a theme during the first visit, but ease of use was a stronger theme during the second visit’’ (p. 9, Gowarty et al., 2021) “Strategies to manage symptoms “[it helps me] redefine my daily thoughts...for people to feel mentally healthy” (p. 11, Kidd et al., 2019) “The most popular PRIME feature was the ability to directly message coaches’’ (p. 1016, Schlosser et al., 2018) “Participants commented on connecting FOCUS to existing structures, including referral services or group meetings’’ (p. 8, Buck et al., 2022) “The advantage of using the app was it, it’s there any time of the today, you can refer to it. It’s like a little notebook you know with exercises and information, just like a little book, you can carry and just refer to it” (Participant 1, p. 728, Taylor et al., 2022) “It was really easy to surf on the website” (Participant 12, Suppl material, Lal et al., 2023) “The fact that there’s an app, and there’s someone like [trial therapist] to like, keep me in tack or like keep checking up on my progress. I think those, those elements made the experience better for me” (Participant 7, p.728, Taylor et al., 2022) “Before I go out, check phone, then leave, pop the bubbles, slow down. I used it every day, not using it now. The tips helpful and personal message when have worries and messages come up, worked as reminder” (Participant L15, p. 689, Greenwood et al., 2022) “The phone actually helps when you’re on the bus … if I start getting agitated about who’s looking at me, and who’s not looking at me yeah, I just start playing with the bubble” (Participant S10, p. 689, Greenwood et al., 2022) “The common hope was that such monitoring may help clinicians, researchers, and scientists to improve treatment programmes and help them feeling better” (p. 5, Zarbo et al., 2022) “If there’s an urgency, we have contacts luke ‘l’autre maison” (Participant 9, Suppl material, Lal et al., 2023) “Approaches that require a range of cognitions such as critical and analytical thinking, evaluating, judging, and weighing options, and deciding on actions that can foreground planning to quit caused participants heightened anxiety and stress’’ (p. 9, Klein et al., 2019) “Maybe visual component more video than are necessary related to the audio” (Participant 17, Suppl material, Lal et al., 2023) “..Some difficulties related to contents and technical features of the mobile application affected the usability and adherence (i.e., text-related questions, bar type considered too distracting, complexity in choosing the correct category for the current activity)” (p. 5, Zarbo et al., 2022) “…It’s quite a lot in a short time. And I feel like for me, it would have been better to do one thing and do it for a few weeks and then add something on” (Participant 6, p. 729, Taylor et al., 2022) “..Umm I did find when I wrote things for the bubbles, the type was a bit big and when the bubbles got smaller I couldn’t read everything it said” (Participant L19, p. 689, Greenwood et al., 2022)
Better awareness and management of mental health	Increased insight; self-management; feelings of empowerment	“Most of the sample reported that they felt that FOCUS helped them manage their symptoms.’’ (p.6, Buck et al., 2022) “The best thing about the app…it makes you feel more positive.’’ (p.834, Lim et al., 2020) “Yeah actually, I can see I am getting better.’’ (Participant 205, p.16, Eisner et al., 2019) “If it’s something could spot my mood going up and down and things it might be useful’’ (p. 15, Eisner et al., 2019) “The sharing of information from the mobile application led to an experience that the therapist had a better understanding of what they were going through’’ (p. 946, Austin et al., 2021) “It was nice to hear people, people’s experiences. It was encouraging to hear, yeah…Um, just how they dealt with the problem…And everyone approached them differently’’ (p. 6, Valentine et al., 2020) “I didn’t understand what recovery was, but now I understand it’s sort of like getting back to normal…like you know, having positive thoughts and things’’ (p. 8, Williams et al., 2018) “I’m not as well as I thought I was’’ (p. 15, Eisner et al., 2019) “A quarter of participants expressed concern that adherence would diminish during relapse’’ (p. 9, Meyer et al., 2018) “Perceived benefits for themselves include the chance to monitor their own emotions and PA, to better understand themselves and to be helped by clinicians” (p. 5, Zarbo et al., 2022) “I don’t worry so much, it’s my neighbours, they make me stressed and then I’ll say, “No, I’ve got to slow down.” You have to, because if not, if you carry on, you make yourself ill and you'll land up in hospital” (Participant L17, p. 689, Greenwood et al., 2022) “I always insisted on going on … a split-second decision…, which is basically fast thinking. I was trained to always look out for the worst case scenario … SlowMo slow thinking wasn’t difficult, but it was different” (Participant O14, p. 689, Greenwood et al., 2022) ‘’It allows the people with the experiences to sort of come up with their own ideas and thoughts’’ (p. 6, Valentine et al., 2020) ‘’A number of young people identified paranoia, social anxiety, and internalized stigma as experiences that interrupted them from being in the Horyzons space’’ (p. 8, Valentine et al., 2020) “It was very hard because I think quickly, but I slowed it down and I’ve learnt how to do that now” (Participant L21, p. 689, Greenwood et al., 2022) “It was daunting at first, cause it was the first time I’d actually spoken about them yeah. The way I felt with my voices. Basically it took me a good 10 min to listen to what she was saying because my voices were telling me not to listen” (Participant S10, p. 688, Greenwood et al., 2022) “There wasn’t like a set routine for sleep until I learned about how a routine could help through the app” (Participant 7, p. 728, Taylor et al., 2022) “In a few cases, among patients, actigraphy monitoring was related to the onset of device-related positive symptoms” (p. 5, Zarbo et al., 2022) ‘’Describing a general sense that they were more aware of and equipped for coping with symptoms in the moment’’ (p. 6, Buck et al., 2022) “I can tell the impact in my everyday life… in helping take care of the girls. Yeah, I mean in my everyday interaction with people, sleep is… It plays a major role, so it's been really good, yeah” (Participant 4, p. 728, Taylor et al., 2022) “Instead of just believing and trusting in that fast-thinking conclusion that I have arrived at, there's been more of an interaction on my part to counteract it with slow thinking… It was much closer to the end of therapy, I was quite actively engaging in slow thinking, quite often” (Participant L18, p. 689, Greenwood et al., 2022) “I never used to go out, you see, and I go out on my scooter now, with confidence. Before I wouldn’t, I always thought people were going to attack me and I don’t feel like that now” (Participant L21, p. 690, Greenwood et al., 2022)
Enhancing communication and relationships	Feeling understood; increased connection; facilitating communication	“Many participants stated tracking created a sense of being understood and created a safe environment.’’ (p.947, Austin et al., 2021) “You know, it’s nice. Like ‘Oh maybe someone cares out there’.’’ (Participant 108, p.9, Gowarty et al., 2021) “Sharing of information strengthened the perceived bond or alliance between them’’ (p. 946, Austin et al., 2021) “Helped me see that ‘you’re not the only one’’’ (p. 1016, Schlosser et al., 2018) “It helped sort of reinforce that some of my experiences and feelings are normal… I’m not a weirdo’’ (p. 6, Valentine et al., 2020) “..Valerie [clinician]: she called me on my cell phone. She’s very nice, She talked half an hour to me. She wanted to make sure I was safe” (Participant 9, Suppl material, Lal et al., 2023) “I found the talks we had …most invaluable. I thought that was amazing …actually having someone to talk to when we were going through it” (Participant O13, p. 688, Greenwood et al., 2022) “He made me really relaxed. Any problems I had, I could talk to him, which I don’t usually … So things came out that I haven’t told anybody'' (Participant L21, p. 688 Greenwood) “… ‘Cause, you like praise the reader with the like ‘well done you've achieved… Like Module 2’, whatever they had, like a well-done sticker, […] it's not like rocket science, so it’s kind of… user-friendly type of thing” (Participant 9, p. 728, Taylor et al., 2022) “Well just watching the videos and erm seeing like the people, I wasn’t alone, because young people like myself or younger than me or older than me does err have mental health, like I’m not the only one” (Participant S1, p. 689, Greenwood et al., 2022) “I felt all of the sections were relevant. Because I felt like the people in the videos felt at some point” (p. 883, Lim et al., 2020) “You’ve really done a lot because you are taking part every day [laughs] just reminding me every day, is part of it. It’s as if we are together!’’ (p. 14, Eisner et al., 2019) “One third emphasized enhancements to the peer-peer feed (e.g., ability to “comment on the feed”, “separate feed into stories, resources, and inspiration”, “improve community outreach and interactivity- get more people on it at the same time’’’ (p. 12, Kidd et al., 2019) ‘’What’s good about it is following each other and giving each other support…they can interact with each other, because it’s important’’ (p. 10, Klein et al., 2019) “Latte and Guilia wanted to “move on” from focusing on their mental illness and did not want to be connected to others who shared this experience’’ (p. 8, Williams et al., 2018) “There was not a lot of people. Nobody online, I did not have a chance to interact” (Participant 14, Suppl material, Lal et al., 2023) “I was hearing other people's like feedback and … some of what they said sort of related to me a little bit…it was just helpful because some of the stuff they were saying sort of, it happened to me before … so, yeah it was, I just kind of relate to it sort of” (Participant L20, p. 689, Greenwood et al., 2022) “I suppose my social life has improved. It's made me more comfortable around people. I’ve met more people and done more things I would say. Like just going to the movies, chilling or playing video games” (Participant L18, p. 690, Greenwood et al., 2022) “Participants, mainly patients, reported increased feelings of being supported and positive affect for supporting others as effect of the weekly monitoring” (p. 5, Zarbo et al., 2022) “When I posted something—wanted other people to answer, not only the moderator” (Participant 14, Suppl material, Lal et al., 2023) “Everyone there has a reason to be there. They’re not judging you, its confidential” (Participant 10, Suppl material, Lal et al., 2023) “I actually think they became more in depth (...) because he sort of knew how I was feeling, too, when I turned up, I didn’t have to spend so much time explaining a lot’’ (p. 947, Austin et al., 2021) “2 mentioned how it encouraged family to be involved and became a focus for education’’ (p. 9, Meyer et al., 2020) “Just being able to talk about my problems and focus on them and just come up with genuine ideas” (Participant L16, p. 688, Greenwood et al., 2022)
Opportunity for reflection	Space to reflect; recovery	“It’s good to actually just ruminate on what is your strengths, like what you’re actually good at.’’ (p.883, Lim et al., 2020) “When I went on to the website, I said to her, “actually I would like some assistance with this, in reflecting on this topic, because I don’t quite understand it and I do need someone to talk to about it.” (p.7, Williams et al., 2018) “Participants spontaneously reported a number of ways in which the app helped them to reflect on their own mental health’’ (p. 14, Eisner et al., 2019) “..You do think everybody is talking about you, I mean that was the big thing that I took from it. It just made me think, ‘Hang on a minute, get a grip, it’s not like that at all.’ That was the biggest thing that I took from it, just seeing things a bit more clearly” (Participant O13, p. 690, Greenwood et al., 2022) “I suppose I looked up to these people on the site and thought, well if other people can, with a mental illness, can be articulate and have a voice and have meaning in their life, well that means perhaps I can too’’ (p. 8, Williams et al., 2018) “[What made the most difference?]’Erm I think the voices of real people. As soon as I left therapy every time, it’s stuck in my head you know. So if I get into that situation I try to rethink it the way that SlowMo taught me really, how to do it” (Participant S7, p. 689, Greenwood et al., 2022)

#### (3) Accessibility

The third theme relates to how easy digital interventions are to use, as well as the opportunity for quicker access to support and being able to assist with potential physical and cognitive impairments. This theme is comprised of two subthemes; (I) easily accessible, and (II) supporting with cognitive/physical difficulties. Having a therapists support in their pocket could be a particularly useful aspect of digital interventions for people with psychosis who may have found it more difficult to access or trust services, and feel able to ask for help. Having symptoms readily recorded within interventions make it easier to recall information to not only share with others, but also in helping to remember experiences they had in the past. This provided participants with more opportunities to learn without having to rely on memory. Being sensitive to literacy and other individual needs is important to ensure that people feel supported and not made to feel that interventions are still inaccessible.

(I) Easily accessible

Participants reported the digital nature of the intervention meant they were able to access this at any time in a range of situations and settings, easily fitting into their daily routine [12, 14, 33–35, 38, 40, 43–45, 47, 48]. ‘’Every day if I go out, I always do what I need to do on it, like take my deep breaths and get me encouraged to go out… that phone is always with me when I’m out. If I stop, I use it as well’’ [Participant L16, p. 689, 12]. Participants reported feeling safer and more supported due to having easy access to healthcare professionals, or through access to coping strategies [12, 35–38, 42, 46, 48]. ‘’Individuals noted that they felt safer or more secure both in regards to their physical safety and in terms of receiving support for mental health issues quickly and easily’’ [p. 8, 35]. Access to, and integration of digital interventions within the routine care that participants were receiving was reported as a key recommendation for future interventions [47].

(II) Supporting with cognitive/physical difficulties

Having a device with them at all times was reported as beneficial for a number of participants who reported difficulties with their memory [40, 44, 45]. ‘’one doesn’t have to sit there and make an effort to remember what to say’’ [p. 946, 33]. Real-time documentation of experiences meant they could record how they were feeling at that time rather than having to rely on their memory, which was particularly useful when experiencing acute symptoms [33, 34, 47]. ‘’Like for me when I was talking about my illness, the more I got ill, the more I couldn’t remember’’ [p. 16, 34].

For participants with lower literacy levels, completing more challenging tasks was reported to be difficult and a barrier to fully engaging with the intervention [34, 38, 43, 48]. Suggestions such as having more visual components or gamifying certain aspects of the intervention was reported to help make it more accessible [48]. ‘’Most effective method is videos’’ [Participant 5, Suppl material, 48]. Recommendations were also made for adaptations for people with visual or hearing impairments, especially for interventions which were delivered on smaller screens [12, 35, 47]. ‘’A way for hearing and vision impaired veterans to be able to use the app’’ [p. 8, 47].

#### (4) Awareness and understanding of mental health

This fourth theme is comprised of three subthemes which include participants' increased insight and understanding of their mental health as well as the impact this had on their self-management behaviours and feelings towards the management of their mental health. These were; (I) increased insight, (II) self-management, and (III) feelings of empowerment. Having increased insight into their mental health helped participants to feel more in touch with their own experiences and feel that they, and others can be better equipped to manage their symptoms. This increases confidence in themselves and others in noticing or intervening at times when relapse may be more likely, therefore taking more control of their mental health rather than it feeling like it is controlling them. Increased symptoms however could interact and impact on participants’ sense of connection towards the intervention and their sense of ability in managing them. At these times, providing participants with additional support in using the intervention could help to increase engagement with it and build hope, confidence and increased skills.

(I) Increased insight

Gaining an increased awareness of symptoms and mood was reported as a key benefit of the digital interventions [12, 14, 33–38, 40, 46, 47]. Understanding the links between symptoms, mood and behaviour, as well as being able to identify factors that could impact mood and symptoms was deemed helpful [33, 34, 36, 38]. *‘’It (the app) has made me more conscious of myself and of what has caused me to feel down and what causes me to feel better’’* [p. 947, 33]. Such information was also reported to be helpful for care providers who could gain an increased insight into the mental health experiences of those they support [33, 45, 46].

Participants reported being able to use the digital interventions to learn new coping strategies and ways to keep well [12, 40, 44, 45]. Information collected over time helped participants to identify factors that could increase the likelihood of relapse [34, 39]. *‘’11 felt that sleep monitoring could be a successful strategy for the early detection of relapse’’* [p. 8, 39]. Reduced levels of engagement with the digital intervention were reported to be useful in indicating relapses [34, 39].

(II) Self-management

Mood and symptom monitoring was reported as useful for being able to better manage symptoms [14, 33, 34, 44, 46, 47]. Having access to resources and strategies enabled participants to support their mental health such as through adhering to medication, or being able to test negative thoughts, voices and paranoid beliefs [12. 34, 35, 37, 47]. *‘’you become your own therapist and that’s what CBT is about, being able to change your behaviour … reassess a situation, about going forward on your own, uhm solution.”* [p. 1078, 14]. Participants reported that a variety of symptoms could impact their ability to self-manage, including both negative and positive symptoms increasing the length of time it took to respond to the intervention, or increasing their avoidance of it [12, 33, 34, 44]. *‘’Several participants described how low mood, negative symptoms, and poor motivation could at times lead them to avoid interacting with the mHealth solution’’* [p. 948, 33]. Factors such as social anxiety and internalised stigma impacted on engagement and therefore on participants ability to engage in self-management behaviours [44].

(III) Feelings of empowerment

Participants reported feeling empowered, motivated, proud and more in control of their mental health as a result of the digital interventions [12, 14, 36, 39 40, 42, 44, 45]. *‘’I think with it being on the phone it’s in your hands a little, it’s under your control a bit more, as opposed to feeling a bit like you’re under house arrest”* [p. 1078, 14]. This helped participants to feel uplifted, happier and more able to engage in day-to-day activities [35, 45, 46, 49]. Engaging in the digital interventions also enabled participants to build their confidence in using coping skills and their own recovery [12, 14, 43, 45–47]. This impacted on participants’ feelings of hope for the future, and for some, reduced feelings of shame, helplessness, and suicidality [42, 45]. *‘’Helped reduce suicidality primarily through instilling some level of hope’’* [p. 1016, 42].

#### (5) Enhancing communication and relationships

In this fifth theme participants’ experiences of feeling understood, connected and better able to communicate with others are discussed. This theme is comprised of three subthemes; (I) feeling understood, (II) increased connection, and (III) facilitating communication. Digital interventions were felt to strengthen existing relationships with professionals through feeling their experiences are understandable, and increased confidence that they can be effectively supported. Reducing feelings of loneliness in relation to their experiences helped to increase feelings of community, knowing that others experience similar things, which may not be as easily accessed in more traditional forms of support. Being supported by a community, as well as supporting others could increase feelings of being cared for, but also may provide people with skills and confidence in helping others and using their experiences to empower themselves and others. Having this community or a sensed need to support others could however result in some feeling pressured to help which could be particularly difficult for those who find interacting with others hard. Knowing what to bring to meetings with professionals may have been a difficult for people in traditional interventions, however with digital interventions participants could more easily instigate conversations and make be open about experiences. This also enabled conversations with families whereby previously it felt difficult to do so.

(I) Feeling understood

Participants reported feeling understood using the digital intervention either due to interactions with a therapist or researcher, others who have experienced psychosis, or just due to the overall understanding and support tone [12, 14, 33, 38, 43, 44, 48]. ‘’many participants continued to perceive both apps as positive and supportive and noted that this was a strength of the apps’’ [p. 9, 36]. This helped participants feel cared and looked out for, which for some enhanced therapeutic relationships [12, 33, 36, 38]. The digital interventions themselves helped participants to recognise that other people also struggle with their mental health, either through being able to interact with peers in the intervention or just as a result of there being an intervention for psychosis [12, 34, 35, 38, 42, 44, 45]. ‘’to be able to see that there were other people out there struggling just as much as I was, or were struggling just as much as I was…was quite helpful’’ [p. 6, 45]. This helped participants to feel less alone and their experiences normalised [12, 34, 38, 42, 44, 45].

(II) Increased connection

The digital interventions increased feelings of connection for participants, through feeling connected with peers, with the person who was accessing their data, and to something bigger [12, 34–38, 42, 44, 49]. ‘’Many expressed that they could relate to others and both understand and feel understood’’ [p. 5, 44]. Having the opportunity to connect with others with shared experiences meant that participants could relate to others and create an environment of reciprocal sharing and support [44–46].

Having easy access to the digital interventions meant that participants could connect with others anywhere they were, which reduced feelings of stigma and loneliness, as well as increased confidence in social situations and feelings of empowerment to support others [12, 35, 38, 44, 45, 48, 49]. ‘’I think I feel a lot more confident in myself… I think prior to it I was a bit, not shy, but a bit hesitant in social situations’’ [p. 883, 49]. However, for some, connecting with others also led participants to feel a sense of responsibility to support others and tiredness from the pressure of offering peer support [36, 44, 45]. ‘’…some people don’t like talking to people like, you know, sometimes you don’t want to talk to people about stuff you might just prefer to deal with an app’’ [Participant 10, p. 728, 43].

(III) Facilitating communication

The digital interventions were reported by participants to facilitate communication with professionals either by using information or data stored in the intervention to help bring up certain topics or to help identify areas for discussion [12, 33–35, 37, 45]. ‘’It keeps me in contact with my mental health professionals out in the community, so it helps [us to] communicate when there is no physical presence’’ [p. 8, 35]. Participants also reported that digital interventions provided them with an easier way to communicate with other people who experience psychosis, and also with their loved ones [39, 44, 49]. The online platform was reported by participants to feel easier and a less threatening means of communicating and opening up about their experiences [34, 35, 43, 45]. ‘’it’s in the privacy of your own home…So there’s no judgement, there’s no judgement of who you are or what you've been’’ [p. 8, 45].

#### (6) Opportunity for reflection

The sixth and final theme is comprised of two subthemes which include digital interventions enabling participants to think and reflect on their mental health, including their ideas on recovery. These were (I) space to reflect, and (II) recovery. Interventions provided participants to stop to pause and notice their experiences. Having experiences or symptoms recorded enabled participants to see these and provide space to rationalise them, providing a broader picture of their experiences. Participants may have previously not had access to references of recovery and how the future may look for them, however the interventions helped to provide a sense of recovery being something that is tangible and realistic.

(I) Space to reflect

The digital interventions included in the reviewed studies were reported by many participants to provide a space to think and reflect on their mental health experiences, either through reviewing daily symptom diaries, hearing others' experiences, or through discussion with clinicians [12, 33, 34, 39, 44–47, 49]. Reflecting on previous and current mental health experiences through accessing data and symptom diaries was reported by some to enable them to look at their experiences from a more objective perspective [33, 34]. ‘’It can be a good thing to just keep an eye on how things are going because it can be a bit like... inside one’s head it’s going splendidly, and then in reality one’s been... (...) or vice versa, if you’re feeling really low but then you realize that... okay, this past week has actually been good’’ [p. 947, 33].

(II) Recovery

Access to information about their own experiences, as well as the experiences of others within the digital interventions was reported by participants to be helpful in getting them to learn and reflect on the meaning of recovery for themselves and others, including increasing beliefs that recovery is possible [12, 34, 36, 45]. ‘’Afterwards I’d just be like “I can get better. I can get better!” there was just, there was this hope that I had when I was using it’’ [p. 16, 34].

## Discussion

This systematic review adds to the limited body of qualitative literature exploring the experiences of people with psychosis using digital interventions. We constructed six themes which showed that the use of digital interventions for psychosis can have a number of benefits, which could be enhanced in existing and future interventions. Some of the findings highlighted across these themes are consistent with reviews both within people with psychosis, but also amongst non-psychosis populations. Specifically, findings related to experiences of technical challenges, concerns regarding privacy and confidentially, a need for training and guidance, as well as interventions being able to enhance therapeutic relationships and provide people with opportunities to learn new skills and connect with others [25, 26, 77–79].

Increasing self-management of symptoms in psychosis is a key feature of digital interventions which can have a positive impact on medication adherence, and also on an individual’s sense of agency over their own symptoms [50]. Enhancing control of their own mental health was reported in the current review as increasing feelings of empowerment and confidence in coping on a day-to-day basis. Empowerment has been identified as one of the key components within recovery, and is particularly important for psychosis populations who are more likely to face disempowering situations [51, 52].

Digital interventions enhancing self-reflection emerged as another theme. Such self-reflection may be helpful in addressing cognitive biases e.g. jumping to conclusions which have been found to be a factor in the onset and maintenance of psychosis [53]. Digital interventions were also reported to help normalise experiences related to psychosis. This process of normalisation can help to reduce stigma in a population that experiences significant rates of stigmatisation compared to other mental health conditions [54]. Further, peer support has been found to have numerous benefits for people with psychosis, including increased feelings of hope, empowerment, optimism and reducing feelings of social isolation and fears of judgement situations [52, 55–57]. Having the option to access peer support and increase connections with others is an important feature of digital interventions and confers many benefits for those using them.

The ability to customise an intervention and receive notifications or reminders was valued. However, study participants also reported on the limited nature of content which could be repetitive over time. Therefore, to ensure longevity of engagement with digital interventions, it may be important to ensure that there is a varied and wide range of content and that individuals have the option to customise the intervention to make it specific to their individual needs. This could help to integrate such interventions into individuals’ everyday lives, which was also reported as beneficial in the current and previous reviews [17, 26].

Easy access to support in daily life could be particularly useful for people with psychosis where there is an increased prevalence of cognitive difficulties [58, 59]. It is also worth considering literacy levels as studies have revealed that these tend to be lower in people with psychosis compared to the general population [60, 61]. Having interventions which are too challenging could create barriers to access as well as increase feelings of shame and stigma, which would likely impact on levels of engagement. Previous research that developed digital interventions with this in mind found that engagement was high and cognitive ability did not impact on participants’ use of the intervention [62].

Consistent with other findings, some of the participants in the reviewed studies reported a lack of experience in using technology limiting engagement with the digital intervention [63, 64]. Compared to the general population, people with psychosis were twice less likely to have foundation or life skills in using digital technology [65]. The opportunity to access training in order to increase confidence and skills in using technology has been suggested as a way to increase accessibility and reduce the digital divide that continues to exist for people with psychosis [64–66]. Further, the use of blended approaches whereby therapists provide support to people whilst using digital interventions has also been found to increase engagement and confidence in using technology [12, 17, 67].

In line with other studies, participants in the reviewed studies raised concerns regarding privacy and worries about being monitored [26, 68]. Participants also reported experiencing technical issues which could cause feelings of frustration, and at times, increased paranoid ideation, which could impact on future engagement. It is worth noting however that raising concerns about, or trust in technology is not mutually exclusive with symptoms such as paranoia and is a consideration in the wider context of digital health care [69] These findings therefore highlight two important points when implementing digital interventions. Firstly, it is crucial to have easy access to ongoing support in order to overcome any technical issues when they arise to reduce disengagement. Secondly, ensuring that individuals using digital interventions are provided with a full explanation and information regarding who can access their data, and the reasons why it is being accessed is vital.

The findings of this review should be considered in the context of the methods in which it was conducted and its limitations. As with all qualitative syntheses, the review is reliant on an analysis of secondary data which was originally generated from different theoretical underpinnings and data analysis methods [70]. People from global majority backgrounds are disproportionately represented within psychosis populations [71–73]. Despite this, there was a lack of reporting of ethnicity in some of the reviewed studies, and in studies that did report on it, the majority of participants identified as White. Therefore, research that reports, and includes people from global majority backgrounds is important to ensure their needs and experiences are represented. The studies within this review explored a wide variety of digital interventions, however the thematic synthesis did not examine potential differences between specific types of digital interventions. This may be useful to investigate in future qualitative reviews as it may highlight specific experiences based on the type of digital intervention used.

The current review was conducted and reported in line with PRISMA guidelines [28]. It is worth nothing, however, that a confidence assessment was not completed as recommended in these guidelines e.g. the GRADE-CERQual approach [76]. Although such assessments have previously been confused with assessments of quality, they are separate and distinct assessments which should be considered in future qualitative systematic reviews [76]. Finally, four different databases were used in order to identify a range of peer-reviewed literature for the review. These databases have been found to be robust in identifying relevant studies for reviews [80]. Research has found that the CINAHL database could also be good source of primary studies in qualitative research and therefore it is recommended that future reviews use this as one of the databases within their search strategy [81]. The current review used a modified version of the PICO, the PICOS, which has been found to be more sensitive to study design and therefore useful in removing irrelevant studies when conducting qualitative systematic reviews [82]. The SPIDER tool has also been recommended for qualitative studies, however research has found the SPIDER may not be as robust as the PICO/PICOS frameworks [29]. Further, the PerSPEcTIF, is another tool developed for qualitative studies however this has not yet been robustly compared against other, more well-established frameworks [83].

The themes in this review can inform stakeholders involved in the development and implementation of digital interventions for people with psychosis. This includes healthcare policy makers, service commissioners, healthcare professionals, and those involved in research. Digital interventions for people with psychosis have a number of benefits for users such as gaining an increased understanding of their experiences, improving self-management of symptoms, and feeling more connected to others. All of these benefits have positive impacts on feelings of empowerment which is an important component of recovery in psychosis and have all been highlighted in this review. However, for those developing and implementing digital interventions for people with psychosis, there is a need to ensure that such interventions are accessible for the population. This includes providing sufficient training and ongoing support to ensure that people with psychosis do not continue to experience a digital divide and can access effective mental health care.

## Data Availability

No datasets were generated or analysed during the current study.
